# Asian medical students’ attitudes towards professionalism

**DOI:** 10.1080/10872981.2021.1927466

**Published:** 2021-05-17

**Authors:** Nirmalatiban Parthiban, Fiona Boland, Darlina Hani Fadil Azim, Teresa Pawlikowska, Marié T. O’Shea, Mohamad Hasif Jaafar, Karen Morgan

**Affiliations:** a Department of Medicine, Hospital Selayang, Selangor, Malaysia; b Royal College of Surgeons in Ireland, Dublin, Ireland; c Perdana University - Royal College of Surgeons in Ireland, Perdana University, Malaysia

**Keywords:** Professionalism, attitude, medical student

## Abstract

**Background:** Professionalism is the basis of trust in patient–physician relationships; however, there is very limited evidence focusing on attitudes towards professionalism among medical students. Hence, the main aim of our study was to investigate Malaysian medical students’ attitudes towards professionalism with specific emphasis on the comparison between pre-clinical and clinical students. Our secondary aim was to compare the differences in perception of medical students in Malaysia (pre-clinical and clinical) with Asian medical students studying in Dublin, Ireland

**Methods:** This study utilized the Professionalism Mini-Evaluation Exercise (P-MEX) instrument which consists of 25 items that represent four skill categories: Doctor–Patient Relationship skills, Reflective skills, Time Management and Inter-Professional Relationship skills. Descriptive statistics were used to describe the demographic information of students and given the ordinal nature of the data, Mann–Whitney U-tests were used.

**Results:** Overall, students have positive attitudes to all the professionalism items with more than 80% of the students agreeing that each of the professionalism attributes is important or very important. There was evidence of a significant difference between Malaysian pre-clinical and clinical students in relation to ‘avoiding derogatory language’ only (p = 0.015). When comparing between Malaysian and Dublin Asian students, there was a statistically significant difference in relation to ‘show interest in patient as a person’ (p < 0.003) for clinical students.

**Conclusion:** Our results point to several curriculum implications such as 1) assessing students’ attitudes towards professional attributes is essential when developing the professionalism curriculum, 2) integrating more effective clinical modules early in the curriculum and 3) considering geographical and cultural factors when assessing perception towards professional attributes.

## Background

Professionalism is the basis of trust in the patient–physician relationship. It has been shown to influence patient care and patient safety, which has now become the cornerstone of high-quality clinical practice [1,2]. Several healthcare organizations such as the American Board of Internal Medicine, the General Medical Council (GMC) and the American Association of Medical Colleges have recognized the paramount importance of professionalism and defined a framework of core elements of professionalism that is expected to be shown by an individual physician in his or her practice [3,4].

There is a growing consensus among medical educationists about the importance of teaching professionalism explicitly during undergraduate years [5,6]. This is reinforced by a study showing the association between unprofessional attitudes in medical school and disciplinary action during practice [7]. In addition, teaching and assessing professionalism in medical school has been shown to have beneficial effects on students and young residents’ professional development [8–10]. For example, a study conducted in Ireland demonstrated an excellent professional output from second-year students from applying a fully integrated professionalism curriculum [9]. Furthermore, teaching professionalism helps in the development of professional identity among medical students and hence it is important to start as part of medical education because it is during this period that transition to professional takes place. Overarching definition of what a medical professional means to the student and addressing the lapses are essential part of the development of the professional identity [11–14].^,^ A systematic review found a significant difference of opinion as to what defines professionalism in the context of medicine [15]. Moreover, in order to accurately define professionalism, geographical location and culture also need to be taken into consideration [16,17]. Sometimes the differences in those factors may lead to differences in behaviour and can be easily perceived as unprofessional. Yet, there is very limited evidence focusing on attitudes towards professionalism among local and international medical students, although it is an essential part of the ongoing effort to give a ‘global’ definition to professionalism.

Therefore, the main aim of our study was to investigate Malaysian medical students’ attitudes towards professionalism with specific emphasis on the comparison between pre-clinical and clinical students. Our secondary aim was to compare the differences in perception of medical students in Malaysia (pre-clinical and clinical) with Asian medical students studying in Dublin, Ireland.

## Methods

A cross-sectional study examining multi-professional undergraduate students’ attitudes to professionalism was conducted between November 2016 and March 2017 in a medical school in Malaysia. A similar study was also completed in a medical school in Dublin and permission was granted to use the international Asian medical students’ data to compare with our participants. The formal curriculum in these two schools is the same. The study was approved by the Institutional Review Board of Perdana University (PUIRBHR0126) and Royal College of Surgeons in Ireland (REC1164)

This study utilized the Professionalism Mini-Evaluation Exercise (P-MEX) instrument to evaluate medical student’s attitude towards professionalism [18]. P-MEX, developed in Canada at McGill University, appears to be a feasible tool with good validity and reliability in evaluating professionalism in clinical training [19]. The data were collected using the P-MEX instrument after obtaining permission for its use from the original author [18]. The P-MEX instrument comprises 25 items that represent four skill categories: Doctor–Patient Relationship skills (8 items), Reflective skills (5 items), Time Management (3 items) and Inter-Professional Relationship skills (9 items). Each item was rated on a 4-points Likert scale as follows: 1 = not at all important 2 = not important 3 = important 4 = very important. The study sample comprised preclinical (foundation year and years 1 and 2) and clinical medical students (years 3, 4 and 5) of academic session 2016/2017. A demographic section was added to the questionnaire to include student’s study year, age, gender, nationality, ethnic group, and if they had another healthcare professional in the family.

In Malaysia, students were approached in groups on campus to complete the survey. The study objectives were explained and study information sheets were distributed. It took approximately 10 minutes to complete the questionnaire. Students were informed explicitly that completion of and the return of the completed questionnaire forms would be considered as consent for their data to be used for analysis. In Dublin, all undergraduate medicine and graduate entry medicine students were invited to take part in this study. Foundation year and years one and two medical students were recruited to take part in this study through distribution of the questionnaire during lectures. Due to the geographical dispersion of undergraduates in years three, four and five of study, who were undertaking clinical placements across city and country, the groups were recruited to take part by email with a link to the online version of the questionnaire. The information gathered was identical regardless of the recruitment route.

Descriptive statistics (counts and percentages) were used to describe the demographic information of students in Malaysia and Dublin. Given the ordinal nature of the data, Mann–Whitney U-tests were used to compare pre-clinical and clinical students in Malaysia and also between Malaysia and Asian medical students studying in Dublin. Due to very few responses, the categories ‘not at all important’ and ‘not important’ were combined for analysis. The Bonferroni correction for multiple comparisons was used and Bonferroni corrected p-values <0.05 were deemed significant. The data were analysed using SPSS Version 23 (IBM Corporation, Armonk, NY) and Stata v15 [20].

## Results

### Characteristics of students

Out of 266 students enrolled in the academic session 2016/2017 in the medical school in Malaysia, 246 students participated, giving a response rate of 92.4%. Table 1 shows the demographic characteristics between the survey respondents from Malaysia and Asian medical students studying in Dublin. In Malaysia, the age of students ranged from 18 to 26 years with a median of 22 (interquartile range 21–23). Among the study participants, 63.8% (n = 157) were female. Most students (67.5%) were from clinical years (Year 3–5). A total of 22.4% (n = 55), 27.6% (n = 68), 41.5% (n = 102) and 6.1% (n = 15) were Malay, Chinese, Indian and others, respectively. Most participants (96.3%) were local students. A total of 39.4% (n = 97) of respondents have a healthcare professional in the family.

**Table 1.** Demographic profiles of the study participants in Malaysia and Asian medical students studying in Dublin

		Malaysia (n = 246)		Dublin (n = 123)	
		**Pre-clinical (n = 80)**	**Clinical** **(n = 166)**	**Pre-clinical** **(n = 101)**	**Clinical** **(n = 22)**
**Gender, % (n)**	Male	37.5 (30)	34.3 (57)	48.5 (49)	50.0 (11)
	Female	61.3 (49)	65.1 (108)	51.5 (52)	50.0 (11)
**Age, median (IQR)**		20.5 (20–21)	22 (22–24)	20 (19–22)	23 (20–23)
**Have a Health Professional in the Family, n (%)**	Yes	33.8 (27)	42.2 (70)	41.6 (42)	50.0 (11)
	No	66.3 (53)	57.8 (96)	58.4 (59)	50.0 (11)

Table 2 presents the percentage of students in Malaysia who have voted the items as not important at all/not important, important or very important. Generally, students have positive attitudes to all the professionalism items with more than 80% of the students agreeing that each of the professionalism attributes is important or very important. The highest percentage of students selecting important or very important was seen for ‘show respect for patient’ (98.8% in pre-clinical and 100% in clinical) and ‘listen actively to patient’ (98.8% in pre-clinical and 100% in clinical). The highest percentage selecting not important at all/not important was seen for ‘accept inconvenience to meet patient’ (11.3% in pre-clinical and 11.5% in clinical) and ‘advocate on behalf of a patient and/or family member’ (15.2% in pre-clinical and 6.6% in clinical).

**Table 2.** The percentage and number of students in Malaysia who perceived the professionalism attribute as not important at all/not important, important or very important

	Percentage of preclinical students who voted %(n)			Percentage of clinical students who voted %(n)			p-value (Bonferroni adjusted)
	Not important at all/Not important	Important	Very Important	Not important at all/Not important	Important	Very Important	
Listen actively to patient	1.3 (1)	5.0 (4)	93.8 (75)	0 (0)	4.2 (7)	95.8 (159)	0.999
Show interest in patient as a person	2.5 (2)	18.8 (15)	78.8 (63)	0 (0)	9.6 (16)	90.4 (150)	0.270a
Show respect for patient	1.3 (1)	3.8 (3)	95.0 (76)	0 (0)	3.0 (5)	97.0 (161)	0.999
Recognize and meet patient needs	1.3 (1)	18.8 (15)	80.0 (64)	1.8 (3)	12.0 (20)	86.1 (143)	0.999
Accept inconvenience to meet patient	11.3 (9)	47.5 (38)	41.3 (33)	11.5 (19)	34.3 (57)	54.2 (90)	0.999
Ensure continuity of patient care	1.3 (1)	23.8 (19)	73.8 (59)	0.6 (1)	20.5 (34)	78.9 (131)	0.999
Advocate on behalf of a patient and/or family member	15.2 (12)	37.5 (30)	46.3 (37)	6.6 (11)	40.4 (67)	53.0 (88)	0.999
Maintain appropriate boundaries with patients/colleagues	1.3 (1)	32.5 (26)	65 (52)	3.0 (5)	23.5 (39)	73.5 (122)	0.999
Demonstrating awareness of limitations	1.3 (1)	28.7 (23)	68.8 (55)	1.2 (2)	25.3 (42)	73.5 (122)	0.999
Admitting errors/omissions	0(0)	20.0 (16)	78.8 (63)	1.8 (3)	17.5 (29)	80.7 (134)	0.999
Soliciting feedback	1.3 (1)	36.3 (29)	61.3 (49)	2.4 (4)	20.5 (34)	77.1 (128)	0.470a
Accepting feedback	0 (0)	22.5 (18)	76.3 (61)	0.6 (1)	18.1 (30)	81.3 (135)	0.999
Maintaining composure in a difficult situation	1.3 (1)	20.0 (16)	77.5 (62)	0.6 (1)	19.3 (32)	77.7 (129)	0.999
Being on time	1.3 (1)	12.5 (10)	85.0 (68)	0.6 (1)	9.6 (16)	89.8 (149)	0.999
Completing tasks in a reliable fashion	0 (0)	23.8 (19)	75.0 (60)	0 (0)	15.1 (25)	84.9 (141)	0.999
Being available to patients or colleagues	3.8 (3)	31.3 (25)	63.7 (51)	1.2 (2)	24.7 (41)	72.9 (121)	0.999
Maintaining appropriate boundaries with patients/colleagues	3.8 (3)	32.5 (26)	62.5 (50)	2.4 (4)	22.3 (37)	75.3 (125)	0.999
Maintaining appropriate appearance	5.6 (4)	30.0 (24)	63.7 (51)	1.8 (3)	19.9 (33)	78.3 (130)	0.468a
Addressing own gaps in knowledge and skills	1.3 (1)	28.7 (23)	68.8 (55)	0.6 (1)	15.1 (25)	84.3 (140)	0.193a
Demonstrating respect for colleagues	0(0)	17.5 (14)	81.3 (65)	0.6 (1)	12.7 (21)	86.7 (144)	0.999
Avoiding derogatory language	1.3 (1)	27.5 (22)	70.0 (56)	0.6 (1)	10.8 (18)	88.6 (147)	0.015a
Assisting a colleague as needed	2.5 (2)	30.0 (24)	66.3 (53)	1.2 (2)	17.5 (29)	81.3 (135)	0.340a
Maintaining patient confidentiality	0(0)	8.8 (7)	90.0 (72)	0.6 (1)	7.8 (13)	91.6 (152)	0.999
Using health resources appropriately	0(0)	18.8 (15)	80.0 (64)	1.2 (2)	22.3 (37)	76.5 (127)	0.999
Respecting rules and procedures of the system	1.3 (1)	20.0 (16)	77.5 (62)	2.4 (4)	12.7 (21)	84.9 (141)	0.999

After Bonferroni correction for multiple comparisons, there was evidence of a significant difference between pre-clinical and clinical students in relation to ‘avoiding derogatory language’ only (p = 0.015). Over 88.6% of clinical students felt that this was very important compared to only 70% of pre-clinical students. Although not significant, a slightly higher percentage of clinical students voted very important for all the items compared to preclinical students except for ‘using health resources appropriately’. Prior to Bonferroni correction significant differences in perception among clinical and pre-clinical students were noted for the following items: ‘show interest in patient as a person’ (p = 0.011), ‘soliciting feedback’ (p = 0.019), ‘maintaining appropriate appearance’ (p = 0.019), ‘addressing own gaps in knowledge and skills’ (p = 0.008), ‘avoiding derogatory language’ (p < 0.001) and ‘assisting a colleague as needed’ (p = 0.014).

Tables 3 and 4 show the comparison of students in Malaysia and Dublin for pre-clinical and clinical years, respectively. Overall, for pre-clinical years, the percentage of students rating an item as important or very important was similar for Malaysian students and Asian students studying in Dublin (Table 3). For both groups ‘show respect for patient’ showed the highest very important percentage (95% in Malaysian students and 92.1% in Asian students studying in Dublin). No statistically significant differences were seen between preclinical students in Malaysia and Dublin.

**Table 3.** Pre-clinical students studying in Malaysia compared to Asian students studying in Dublin

	Percentage Malaysia students who voted (pre-clinical) %(n)			Percentage of Dublin students who voted (pre-clinical) %(n)			p-value (Bonferroni adjusted)
	Not important at all/Not important	Important	Very Important	Not important at all/Not important	Important	Very Important	
Listen actively to patient	1.3 (1)	5.0 (4)	93.8 (75)	0.6 (1)	9.4 (4)	90.1 (75)	0.999
Show interest in patient as a person	2.5 (2)	18.8 (15)	78.8 (63)	5.0 (2)	21.8 (15)	73.3 (63)	0.999
Show respect for patient	1.3 (1)	3.8 (3)	95.0 (76)	0 (0)	7.9 (8)	92.1 (93)	0.999
Recognize and meet patient needs	1.3 (1)	18.8 (15)	80.0 (64)	0 (0)	21.8 (22)	78.2 (79)	0.999
Accept inconvenience to meet patient	11.3 (9)	47.5 (38)	41.3 (33)	19.8 (20)	38.6 (39)	41.6 (42)	0.999
Ensure continuity of patient care	1.3 (1)	23.8 (19)	73.8 (59)	0 (0)	263 (26)	73.7 (73)	0.999
Advocate on behalf of a patient and/or family member	15.2 (12)	37.5 (30)	46.3 (37)	8.1 (8)	50.5 (50)	41.4 (41)	0.999
Maintain appropriate boundaries with patients/colleagues	1.3 (1)	32.5 (26)	65.0 (52)	3.1 (3)	31.6 (31)	65.3 (64)	0.999
Demonstrating awareness of limitations	1.3 (1)	28.7 (23)	68.8 (55)	2.0 (2)	33.3 (33)	64.6 (64)	0.999
Admitting errors/omissions	0 (0)	20.0 (16)	78.8 (63)	3.0 (3)	18.2 (18)	78.8 (78)	0.999
Soliciting feedback	1.3 (1)	36.3 (29)	61.3 (49)	3.1 (3)	36.7 (36)	60.2 (59)	0.999
Accepting feedback	0 (0)	22.5 (18)	76.3 (61)	2.0 (2)	20.2 (20)	77.8 (77)	0.999
Maintaining composure in a difficult situation	1.3 (1)	20.0 (16)	77.5 (62)	1.0 (1)	17.5 (17)	81.4 (79)	0.999
Being on time	1.3 (1)	12.5 (10)	85.0 (68)	2.0 (2)	9.1 (9)	88.9 (88)	0.999
Completing tasks in a reliable fashion	0 (0)	23.8 (19)	75.0 (60)	5.1 (5)	17.2 (17)	77.8 (77)	0.999
Being available to patients or colleagues	3.8 (3)	31.3 (25)	63.7 (51)	7.1 (7)	36.7 (36)	56.1 (55)	0.999
Maintaining appropriate boundaries with patients/colleagues	3.8 (3)	32.5 (26)	62.5 (50)	4.0 (4)	29.3 (29)	66.7 (66)	0.999
Maintaining appropriate appearance	5.6 (4)	30.0(24)	63.7 (51)	4.0 (4)	33.3 (33)	62.6 (62)	0.999
Addressing own gaps in knowledge and skills	1.3 (1)	28.7 (23)	68.8 (55)	3.0 (3)	15.2 (15)	81.8 (81)	0.999
Demonstrating respect for colleagues	0 (0)	17.5 (14)	81.3 (65)	0 (0)	16.2 (16)	83.8 (83)	0.999
Avoiding derogatory language	1.3 (1)	27.5 (22)	70.0 (56)	3.1 (3)	20.4 (20)	76.5 (75)	0.999
Assisting a colleague as needed	2.5 (2)	30.0 (24)	66.3 (53)	2.0 (2)	29.3 (29)	68.7 (68)	0.999
Maintaining patient confidentiality	0 (0)	8.8 (7)	90.0 (72)	0 (0)	8.1 (8)	91.9 (91)	0.999
Using health resources appropriately	0 (0)	18.8 (15)	80.0 (64)	2.0 (2)	16.2 (16)	81.8 (81)	0.999
Respecting rules and procedures of the system	1.3 (1)	20.0 (16)	77.5 (62)	1.0 (1)	25.3 (25)	73.7 (73)	0.999

**Table 4.** Clinical students studying in Malaysia compared to Asian students studying in Dublin

	Percentage Malaysia students who voted (clinical) %(n)			Percentage of Dublin students who voted (clinical) %(n)			p-value (Bonferroni adjusted)
	Not important at all/Not important	Important	Very Important	Not important at all/Not important	Important	Very Important	
Listen actively to patient	0 (0)	4.2 (7)	95.8 (159)	0(0)	18.2(4)	81.1 (18)	0.223a
Show interest in patient as a person	0 (0)	9.6 (16)	90.4 (150)	4.5 (1)	36.4 (8)	59.1 (13)	<0.003a
Show respect for patient	0 (0)	3.0 (5)	97.0 (161)	0 (0)	4.5 (1)	95.5 (21)	0.999
Recognize and meet patient needs	1.8 (3)	12.0 (20)	86.1 (143)	4.5 (1)	9.1 (2)	86.4 (19)	0.999
Accept inconvenience to meet patient	11.5 (19)	34.3 (57)	54.2 (90)	22.7 (5)	50.0 (11)	27.3 (6)	0.363a
Ensure continuity of patient care	0.6 (1)	20.5 (34)	78.9 (131)	0 (0)	27.3 (6)	72.7 (16)	0.999
Advocate on behalf of a patient and/or family member	6.6 (11)	40.4 (67)	53.0 (88)	13.6 (3)	36.4 (8)	50.0 (11)	0.999
Maintain appropriate boundaries with patients/colleagues	3.0 (5)	23.5 (39)	73.5 (122)	9.1 (2)	18.2 (4)	72.7 (16)	0.999
Demonstrating awareness of limitations	1.2 (2)	25.3 (42)	73.5 (122)	4.5 (1)	18.2 (4)	77.3 (17)	0.999
Admitting errors/omissions	1.8 (3)	17.5 (29)	80.7 (134)	0 (0)	18.2 (4)	81.8 (18)	0.999
Soliciting feedback	2.4 (4)	20.5 (34)	77.1 (128)	4.5 (1)	31.8 (7)	63.6 (14)	0.999
Accepting feedback	0.6 (1)	18.1 (30)	81.3 (135)	0 (0)	22.7 (5)	77.3 (17)	0.999
Maintaining composure in a difficult situation	0.6 (1)	19.3 (32)	77.7 (129)	0 (0)	22.7 (5)	77.3 (17)	0.999
Being on time	0.6 (1)	9.6 (16)	89.8 (149)	4.5 (1)	13.6 (3)	81.8 (18)	0.999
Completing tasks in a reliable fashion	0 (0)	15.1 (25)	84.9 (141)	0 (0)	9.1 (2)	90.1 (20)	0.999
Being available to patients or colleagues	1.2 (2)	24.7 (41)	72.9 (121)	13.6 (3)	22.7 (5)	63.6 (14)	0.999
Maintaining appropriate boundaries with patients/colleagues	2.4 (4)	22.3 (37)	75.3 (125)	9.1 (2)	18.2 (4)	72.7 (16)	0.999
Maintaining appropriate appearance	1.8 (3)	19.9 (33)	78.3 (130)	4.5 (1)	40.9 (9)	54.5 (12)	0.368a
Addressing own gaps in knowledge and skills	0.6 (1)	15.1 (25)	84.3 (140)	4.5 (1)	22.7 (5)	72.7 (16)	0.999
Demonstrating respect for colleagues	0.6 (1)	12.7 (21)	86.7 (144)	0 (0)	13.6 (3)	86.4 (19)	0.999
Avoiding derogatory language	0.6 (1)	10.8 (18)	88.6 (147)	0 (0)	13.6 (3)	86.4 (19)	0.999
Assisting a colleague as needed	1.2 (2)	17.5 (29)	81.3 (135)	9.1 (2)	22.7 (5)	68.2 (15)	0.999
Maintaining patient confidentiality	0.6 (1)	7.8 (13)	91.6 (152)	0 (0)	4.5 (1)	95.5 (21)	0.999
Using health resources appropriately	1.2 (2)	22.3 (37)	76.5 (127)	0 (0)	18.2 (4)	81.8 (18)	0.999
Respecting rules and procedures of the system	2.4 (4)	12.7 (21)	84.9 (141)	0 (0)	13.6 (3)	86.4 (19)	0.999

aaaa

For clinical years (Table 4), for most items a higher (or similar) percentage of students in Malaysia selected very important compared to Dublin students. After Bonferroni adjustment, there was a statistically significant difference in relation to ‘show interest in patient as a person’ (p < 0.003). In Malaysia 90.4% of students selected ‘very important’ compared to 59.1% of Dublin students. Prior to Bonferroni correction, the following items also showed a statistically significant difference between Malaysia and Dublin, with a higher number of Malaysian students selecting very important: ‘listen actively to patient’ (p = 0.009), ‘accept inconvenience to meet patient’ (p = 0.015) and ‘maintaining appropriate appearance’ (p = 0.015).

## Discussion

P-MEX items were developed by selecting 25 behaviours that reflected most of the professionalism attributes identified at the McGill workshop [18]. The identified attributes were similar to those recommended by the National Board of Medical Examiners and the Association of American Medical Colleges. Hence, all behaviours listed on the P-MEX questionnaire are important parts of medical professionalism. By knowing the extent of knowledge and attitude towards each behaviour, medical educators can determine if the students’ definition of professionalism is congruent with the definition provided by the international organizations. However, understanding the students’ attitudes towards professionalism has never been an easy task, further challenged by the person’s context, geographical region and culture [16,17].

In our study, most students from Malaysia felt ‘Show respect for patient’ was very important trait of professionalism (96.3%). This finding was similar to other studies conducted in Malaysia [21,22]. This may reflect the success of the current curriculum of the school. ‘Show respect to patient’ is frequently invoked in both pre-clinical and clinical teaching. It is taught in several forms including respect for autonomy, respect for human life and dignity and respect for patient privacy and confidentiality. Many studies including a systematic review showed that clinical tutors and hospital specialists played an important role in enhancing students’ attitude towards the professional attribute during clinical training through demonstration and role modelling [23,24]. On the other hand, least percentage of students felt ‘Accept inconvenience to meet patient’ as very important part of professionalism (Malaysia-50.8%). These concordances with several studies which highlighted that the concept of subordinating self-interest appears less appealing to students as a professional attribute [25,26]. A timely literature review by O’Riordan.C highlighted on how self-interest issues work as potent force in the modern world producing great stress on the practice of medicine [27]. Besides, Ginsburg, Regher and Lingard also reported that actions suggested by students for professional dilemmas were often framed by their own self-interest [28]. Such findings suggest that students may have a different perception on certain aspects of professionalism and warrants more explicit teaching of the poor professional attributes during interactions in the classroom.

In general, the clinical group had a higher percentage of students voting for most items as a very important part of professionalism as compared to pre-clinical students. This finding is similar to a study conducted in Malaysia which reported that clinical students expressed better attitude towards professionalism attributes as compared to the pre-clinical group though the differences reported were not significant [21]. Our findings suggest that students’ exposure to patient care does not only enhance the overall development of professionalism but also improve the manner the students speak and write about patient by avoiding derogatory language. This gives us an important curriculum implication. It necessitates the need to introduce clinical modules early during the pre-clinical years to improve students’ overall attitude towards professionalism. Nishigori et al. reported that introduction of international electives helped the students to learn professional values better through reflection and observation [29]. Similarly, Parkin and Shin (2001) reported a successful outcome from introducing ‘illness module’ to the new medical students [30]. This study found that the least percentage of clinical students in Malaysia viewed ‘Accept inconvenience to meet patient’ (54.2%) and ‘Advocate on behalf of a patient and/or family member’ (53%) as very important part of professionalism. On the other hand, least percentage of clinical students in Dublin perceived ‘Accept inconvenience to meet patient’ (77.3%) as very important part of professionalism. With increasing years of training, several studies suggested that knowledge and practices could be different. Although the students received formal training in the conceptual aspects of professionalism, they also needed help in negotiating some of the challenges to medical professionalism that were encountered in clinical settings [13]. Understanding future excessive work load, sleep deprivation, and constant access demands make the students and physicians value their free time. They would try to avoid meeting the patients if they have increased risk for burnout such as covering of a departing physician [30].

The movements of medical students and qualified health practitioners across the globe have further challenged attempts at defining and assessing professionalism. As previous studies explored the influence of geographical and cultural variations on professionalism, Jha et al. timely pointed out that when exploring the culture pertaining to any interactions, whether it is teacher-student or doctors–patient, it is essential to focus on the subject’s values, belief and background [31]. This is on the basis that culture represents the values and beliefs expressed by the individual subject, which often becomes central to the perception and development of professionalism [31].

It is worth noting that there were differences of opinion for certain professional attributes among students in Malaysia and Asian students in Dublin. Although this could be partly due to the higher number of clinical students among the Malaysian sample, we noted an almost similar trend when we carried out independent comparisons between the clinical and preclinical students. It was interesting to note “Show interest in patient as a person” professionalism trait was significantly lower among Asian students in Dublin. This may point out to the possibility of cultural/language variations affecting nonverbal communication (to convey sense of warmth, empathy, caring and reassurance) with patients of different culture/background. Our finding warrants further research to compare the attitudes towards professionalism between international students and local Dublin students in order to provide evidence on the effect of cultural variations on professional attributes. Apart from cultural and geographical variations, these findings also warrant for further research to see the influence of ethnicity on the professional attributes.

Besides providing evidence for better patient–physician relationship, such comparison studies will also help to determine if there are any conflicting values among the international students. This is because conflicting values of an individual can have implications on their well-being, as described by Lu et al. in their ‘Culture fit’ theory [8]. The author described the potential adverse effect on student’s psychosocial and well-being if the students’ original values and belief conflicts with values of the host country. There has been substantial work focused on interventions to support International Medical Graduates to make a successful transition to their host country, but there seems to be a lack of such evidence for international medical students [32,33].

## Limitations

As this is a cross-sectional study, a single assessment of the students’ attitude towards professionalism attributes will not confirm that these attitudes would translate into behaviours in the future. There is also a noticeable difference in the ratio of female and male participants owing to the greater number of enrolled female students in all batches. We included only international Asian students in Dublin, which limits the ability to generalize the results for a wider population. Despite these limitations, some interesting trends were found which highlight the need for further research. In addition to the recommendations above, future research may also look into the cause of the perceptional differences and perhaps include the perception or expectations of other stakeholders such as the faculty members, other health-care professionals, patients and public in order to refine the definition of professionalism.

## Conclusions

Our results point to several curriculum implications. Our study showed it is essential to assess students’ attitudes towards professional attributes to allow medical educators to update the professional courses and tailor them to meet the needs of the students. This will further help to contextualize the professionalism lapses. Furthermore, we also proposed the integration of more effective clinical modules early in the curriculum. To the best of our knowledge, this would be the second but most recent study investigating students’ attitudes towards professionalism attributes from two culturally and geographically distinct groups [34]. Our findings favours the importance of considering geographical and cultural factors when assessing perception towards professional attributes.

## Practice points

1. Differences in perception towards professional attributes do exist among medical students
2. It’s important to assess student’s attitudes towards professional attributes through evidence-based approaches when developing curriculum for professionalism.
3. Curriculum for professionalism should be tailored to leverage the least favourite professional traits.
4. Educators should integrate more effective clinical modules, especially during the preclinical years.
5. Geographical and cultural factors may result in a variation of perception towards professional attributes and should be considered when developing the curriculum for professionalism.
